# Traumatic avulsion of the anterior medial meniscus root combined with PCL injury: a case report

**DOI:** 10.1186/s12891-020-03671-x

**Published:** 2020-09-30

**Authors:** Anqi Wang, Hongzhang Lu

**Affiliations:** Department of Orthopedics, Peking University First Hospital, No.8 XiShiku Street, XiCheng District, Beijing, 100034 China

**Keywords:** Meniscus root avulsion, PCL injury, Meniscal repair, Suture anchor

## Abstract

**Background:**

Avulsion of the anterior medial meniscus root (AMMR) has a low incidence rate, especially when it is combined with posterior cruciate ligament (PCL) injury, which hasn’t been reported in any literature to date. The aim of this study was to share our experience in the diagnosis and treatment of a patient with traumatic avulsion of AMMR combined with PCL injury.

**Case presentation:**

This article reports a 26-year-old male patient diagnosed with traumatic avulsion of the AMMR with PCL injury. After arthroscopic surgery, he achieved remission of symptoms and recovery of functions.

**Conclusions:**

Anterior meniscus root injuries are relatively rare. Its diagnosis can be made preliminarily based on clinical manifestations, physical examinations, and magnetic resonance imaging (MRI), and then confirmed by arthroscopic exploration. Arthroscopic suture anchor fixation of the injured anterior meniscus horn shows a good therapeutic effect.

## Background

Meniscus tear, a common condition in young and middle-aged adults, has a mean annual incidence of 9.0 per 10,000 in men, and 4.2 per 10,000 in women [1]. The avulsion of the posterior medial meniscus root (PMMR) occurs more often than that in the AMMR, and therefore most of the available studies have focused on PMMR avulsion [2–5]. So far, only a few cases of AMMR avulsion have been reported that were caused by iatrogenic factors [6], or anatomical variation and combined with anterior cruciate ligament (ACL) injury [7, 8]. No reports of AMMR avulsion combined with PCL injury have been found yet. In this case study, we report a 26-year-old man with traumatic avulsion of the AMMR complicated with PCL injury. The purpose of this study was to share our experience in the diagnosis and treatment of this rare condition.

## Case presentation

A 26-year-old male patient was referred to our clinic for pain in the right knee while walking, and being unable to run or jump for 6 weeks, after a jump landing on flat ground. Since the injury, he had received immobilization of the injured knee in extension position for 4 weeks with a hinged knee brace and crutches, then tried flexion and loading weight without the brace for 2 weeks, which however, did not relieve the pain effectively. Physical examinations on admission showed effusion of the injured knee, tenderness in the anteromedial aspect of the knee, and deep anterior knee pain when the flexion angle was greater than 40°. In addition, a positive McMurray test and grade I° posterior instability were found without unstable collateral ligaments.

**Fig. 1:**
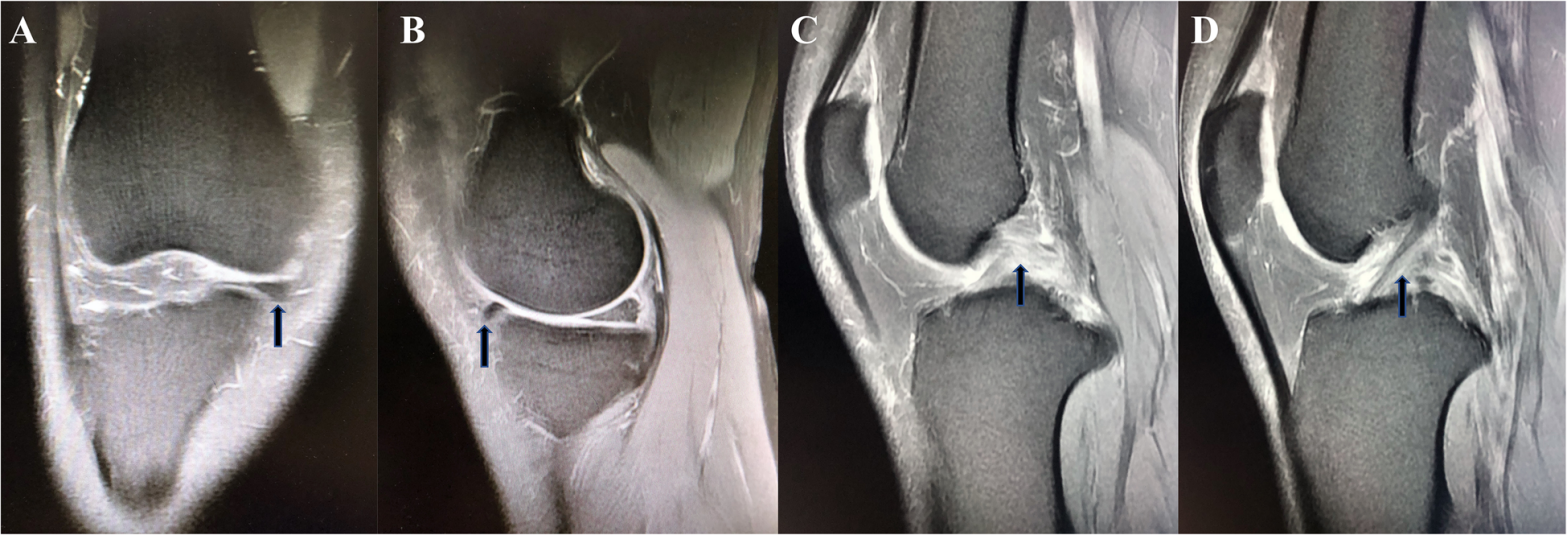
T2-weighted MRI of the injured knee joint **a** Coronal view showed protrusion of the anterior medial meniscus horn (*arrow*). **b** Sagittal view showed antedisplacement of the anterior medial meniscus horn (*arrow*), **c** and high signal of the PCL (*arrow*), **d** while the ACL was normal (*arrow*)

Coronal view of MRI showed protrusion of medial meniscus at the front of the injured knee (Fig. 1a), and sagittal view showed antedisplacement (Fig. 1b) of the anterior medial meniscus horn, combined with high T2 signal in the PCL (Fig. 1c), while the ACL was normal (Fig. 1d).

Based on the patient’s medical history and findings of the physical and radiographic examinations, traumatic avulsion of the AMMR combined with PCL injury was highly suspected. Hence, we recommended arthroscopic exploration and essential surgical interventions to the patient.

**Fig. 2:**
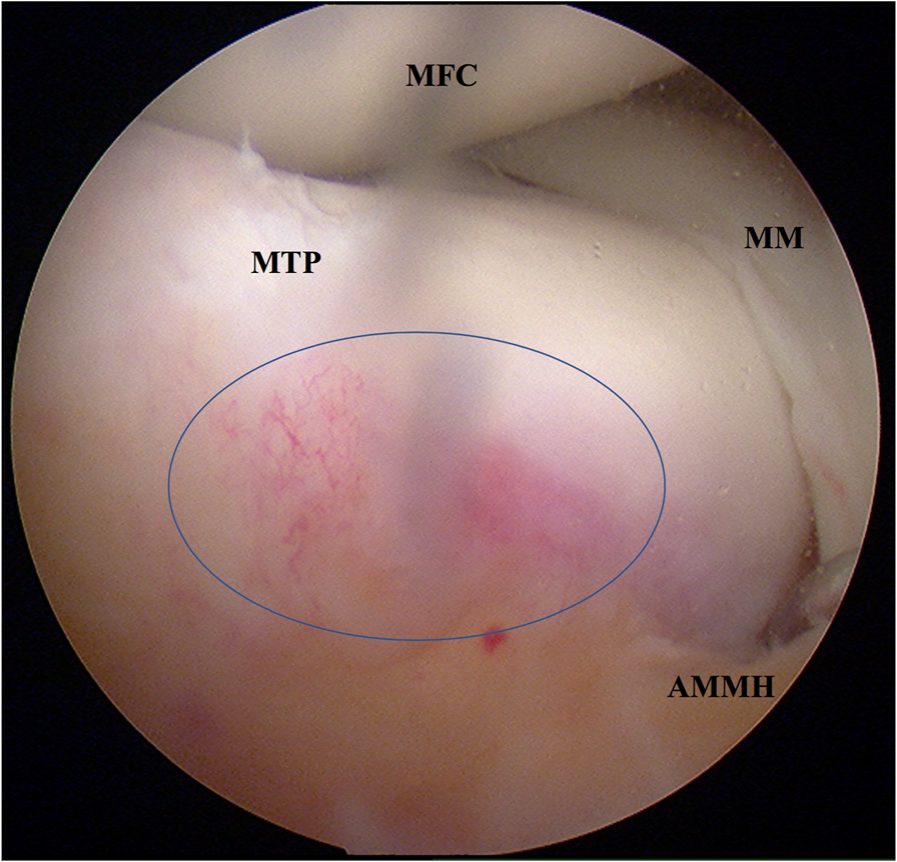
Diagnostic arthroscopy via a standard anterolateral portal confirmed the diagnosis. Arthroscopy showed detachment of the anterior horn of the medial meniscus, and bruise and hyperemia on the downward slope from the medial articular plateau to the intercondylar region (*blue oval*). *MFC:* medial femoral condyle; *MTP:* medial tibial plateau; *MM:* medial meniscus; *AMMH:* anterior medial meniscus horn

Arthroscopic examination found mild synovial hyperplasia and pannus formation, without obvious injury or degeneration of the articular cartilage. The ACL was intact and the tension was normal. Hyperemia and partial PCL injury but proper tension was observed. Furthermore, we discovered a detached anterior horn of the medial meniscus, and bruise and hyperemia on the downward slope from the medial articular plateau to the intercondylar region (Fig. 2).

**Fig. 3:**
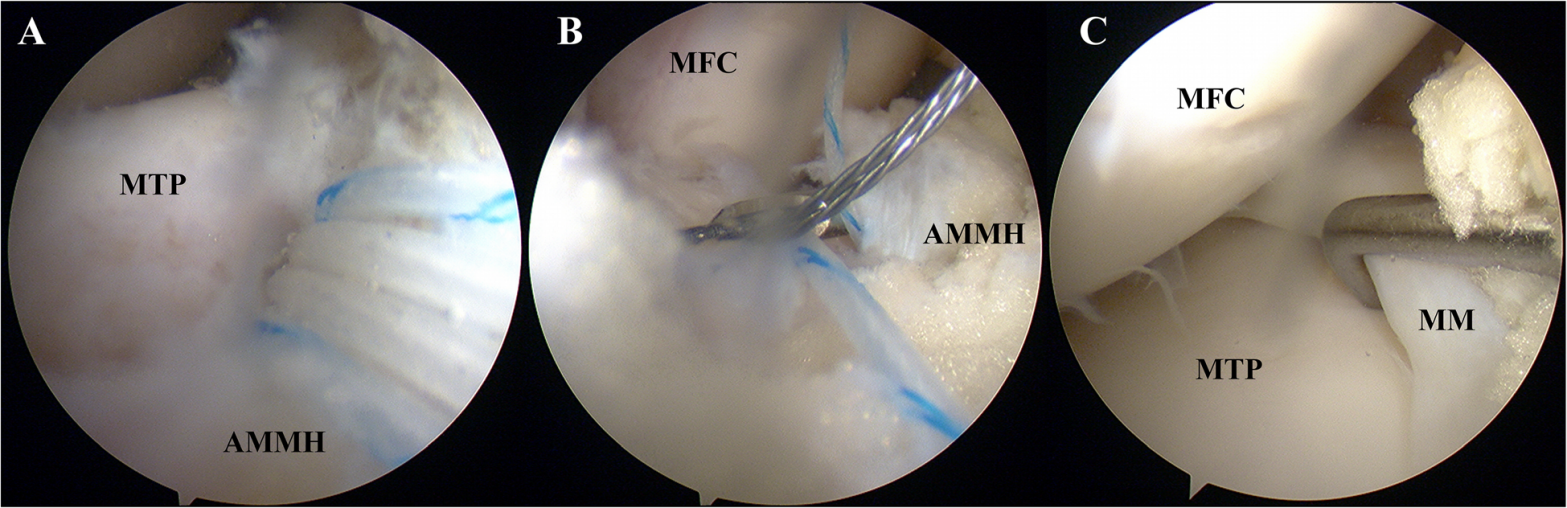
Arthroscopic suture anchor fixation. **a** A suture anchor (TwinFix Ti 3.5 mm, Smith & Nephew, Memphis, TN, US) with four sutures’ free ends was inserted into the bruise area. **b** Sutures’ free ends of the inserted anchor were hooked penetrating the anterior horn of the medial meniscus with a curved suture hook. **c** The anterior medial meniscus horn covered the marginal cartilage of the tibial plateau well; increased stability and decreased mobility were observed after the fixation. *MFC:* medial femoral condyle; *MTP:* medial tibial plateau; *MM:* medial meniscus; *AMMH:* anterior medial meniscus horn

Arthroscopic suture anchor fixation of the injured anterior meniscus horn was performed. After clearing the hyperplastic synovium and pannus, a suture anchor (TwinFix Ti 3.5 mm, Smith & Nephew, Memphis, TN, US) was inserted into the bruise area of the downward slope from the medial articular plateau to the intercondylar region through the medial portal. Subsequently, four free ends of the sutures of the inserted anchor were hooked penetrating from the inferior to superior surface of the anterior horn of the medial meniscus with a curved suture hook. The penetrating points were placed at a distance 1.0 cm apart. Finally, two mattress-suture stitches were made with a knotter to make the torn anterior medial meniscus horn reattach the tibial plateau. An increased stability and decreased mobility of the anterior horn of the medial meniscus were then confirmed under arthroscopy, covering the marginal cartilage of the tibial plateau well (Fig. 3). No surgical intervention was taken to the PCL considering its proper tension. The surgical wound was then closed by layers after a thorough flushing of the articular cavity with saline and careful hemostasis with plasm radiofrequency.

The patient received physical rehabilitation after the surgery. Swelling of the operated knee joint could be controlled with ice and compression in the first 2 weeks after the operation. The knee was immobilized in an extension position with a hinged knee brace for 4 weeks after surgery. Flexion was allowed up to 90° since week 4 to 6 postoperation, then up to 120° since week 6 to 8, and finally reached to the maximum flexion since week 8 to 12. The loading of operated knee was allowed partial incremental weight with crutches from week 4 to 9 postoperation, and a full-weight loading was permitted starting from week 10 to 12. Quadriceps activation was encouraged in the first 2 weeks after the surgery and muscle strengthening was necessary afterwards.

The patient had prominent pain relief and functional recovery in 12 weeks postoperatively. Physical examination found neither tenderness nor a positive McMurray test, and the posterior instability disappeared 12 weeks after the operation.

## Discussion and conclusions

To our knowledge, this is the first report describing a traumatic avulsion of the AMMR combined with PCL injury. Comparing to posterior meniscus root injury, fewer cases of anterior meniscus root injury have been reported due to its low incidence rate. In 2007, Navarro-Holgado and colleagues [8] reported two cases of anterior medial meniscus detachment associated with tearing of the ACL, to which the anatomic variation of the AMMR attachment sites was thought to have contributed. Both of the patients received arthroscopic partial meniscectomy and ACL reconstruction. In 2011, Toy et al. [7] reported a case of combined avulsion of the ACL and the anterior lateral meniscus root in a 14-year-old boy. And then, in 2015, Feucht MJ et al. [6] reported a case of simple iatrogenic avulsion of the AMMR, which were innovatively repaired by an arthroscopic suture anchor fixation of the injured anterior meniscus horn, leading to satisfactory therapeutic effects.

When the knee joint is at a flexion position, the posterior meniscus horn maintains the internal rotation stability, and the anterior meniscus horn functions for the external rotation stability of the joint. When the flexion angle is less than 30°, the anterior medial meniscus horn withstands a high shear strength and prevents the femur from antedisplacement [9, 10]. PCL can prevent retrodisplacement of the tibia [11], meanwhile, the anterior medial meniscus horn can prevent antedisplacement of the femur [12], the two coordinately prevent the excessive motion of the knee joint. In our case, however, the PCL couldn’t bear the tension caused by the excessive motion of the knee joint, and thus a high shear was exerted to the anterior medial meniscus horn, leading to traumatic avulsion of the AMMR complicated with PCL injury.

Preoperative diagnosis of AMMR avulsion is difficult because of its low morbidity and inadequate diagnostic criteria [13, 14], plus the anatomic variations of the AMMR. The types of the anatomic variation of the anterior medical meniscus roots have been controversial. Berlet et al. [15] described four types of bony tibial insertions of the AMMR. Type I locates on the flat intercondylar region of the tibial plateau (59%); Type II on the downward slope from the medial articular plateau to the intercondylar region (24%); Type III on the anterior slope of the tibial plateau (15%); and Type IV locates on no firm bony insertion (2%). Some researchers, however, believed that the Type IV did not really exist [16]. Intact structure of the meniscus can buffer the pressure in the knee joint and thus prevent extrusion of the meniscus [17, 18]. In this case study, the patient had explicit trauma history of the knee and non-ideal conservative treatment outcome for pain relief. His physical examination showed tenderness of the anteromedial knee region and a positive McMurray test, MRI disclosed protrusion and antedisplacement of the anterior medial meniscus horn, which revealed potential avulsion of the AMMR. Arthroscopic examination found extrusion and instability of the anterior medial meniscus horn, as well as bruise and hyperemia on the downward slope from the medial articular plateau to the intercondylar region, which confirmed the diagnosis.

**Fig. 4:**
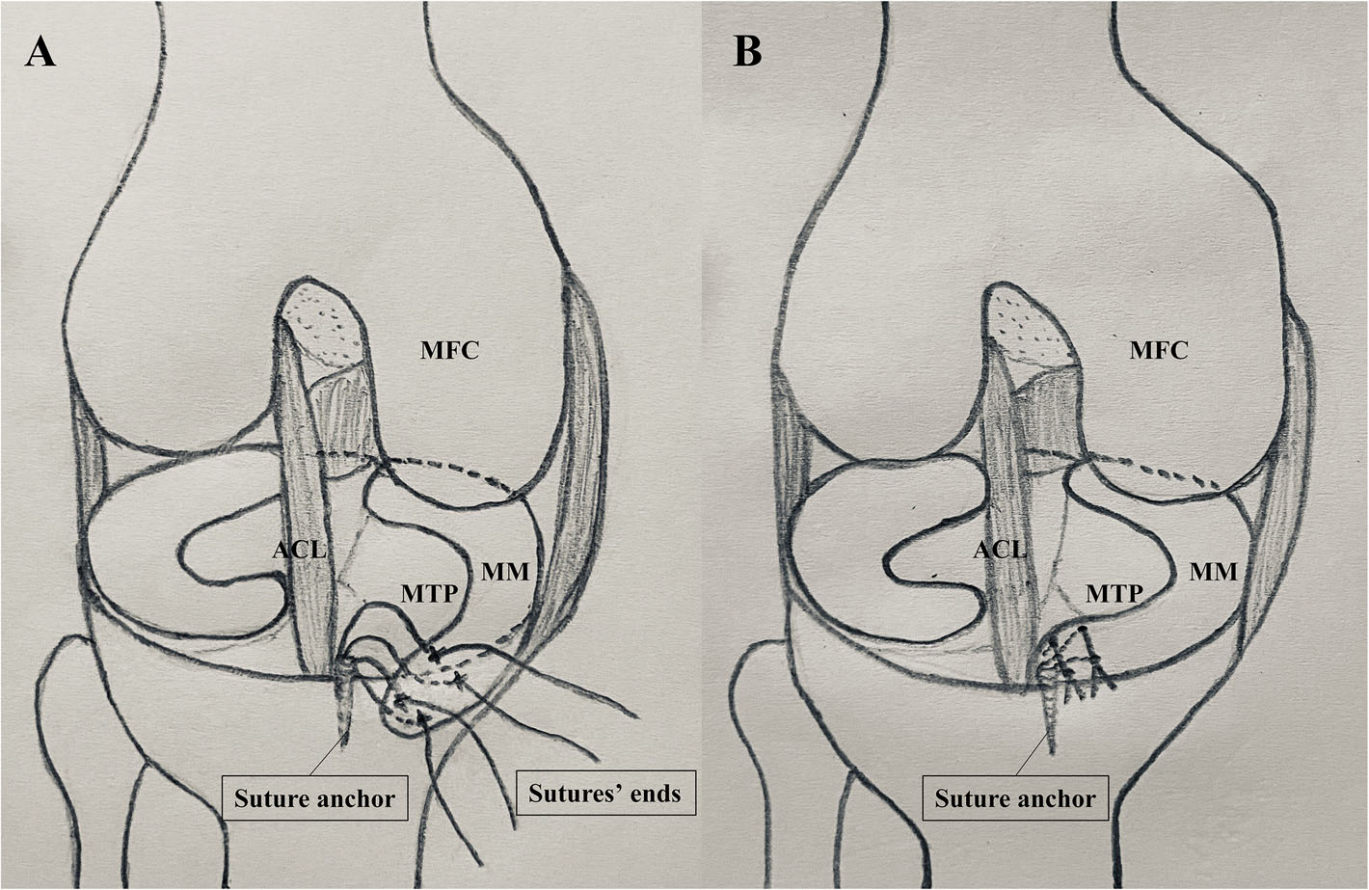
Diagrammatic drawing of suture anchor fixation. **a** A suture anchor with four sutures’ free ends was inserted into the bruise area, and sutures’ free ends were hooked penetrating the anterior horn of the medial meniscus. **b** Two mattress-suture stitches were made to make the torn anterior medial meniscus horn reattached the tibial plateau. *MFC*: medial femoral condyle, *MTP:* medial tibial plateau, *MM:* medial meniscus, *ACL:* anterior cruciate ligament

With the technique introduced by Feucht MJ et al. [6], we performed arthroscopic suture anchor fixation of the injured anterior meniscus horn on our patient. A suture anchor was planted into the bruise area of downward slope from the medial articular plateau to the intercondylar region through the medial portal, and two mattress-suture stitches were made to reattach the anterior medial meniscus horn (Fig. 4). Although preoperative physical examination showed grade I° posterior instability, and sagittal T2WI of MRI found high signal in the PCL, arthroscopic exploration revealed hyperemia and partial injury but proper tension of the PCL, which in our opinion, indicated that a surgical treatment was not necessary. The preoperative grade I° posterior instability might be caused by the partial injury of the PCL, which might also result from the AMMR avulsion. After postoperative rehabilitation, the PCL were self-cured and the AMMR were fixed, leading to a pain relief, negative McMurray test, and posterior stability.

In conclusion, anterior meniscus root injury can be diagnosed preliminarily with clinical manifestations and MRI, and then confirmed by arthroscopy. Arthroscopic suture anchor fixation of the injured anterior meniscus horn has a good therapeutic effect.

## Data Availability

All data generated or analyzed during this study are included in this article.

## Abbreviations

AMMR: Anterior medial meniscus root
PMMR: Posterior medial meniscus root
PCL: Posterior cruciate ligament
ACL: Anterior cruciate ligament
MRI: Magnetic resonance imaging

## References

[1] Cavanaugh JT (2014). Rehabilitation of meniscal injury and surgery. J Knee Surg.

[2] Bernard CD, Kennedy NI, Tagliero AJ, Camp CL, Saris DBF, Levy BA, Stuart MJ, Krych AJ (2020). Medial meniscus posterior root tear treatment: a matched cohort comparison of nonoperative management, partial Meniscectomy, and repair. Am J Sports Med.

[3] Kim JY, Bin SI, Kim JM, Lee BS, Oh SM, Cho WJ, Lee JH. Partial meniscectomy provides the favorable outcomes for symptomatic medial meniscus tear with an intact posterior root. Knee Surg Sports Traumatol Arthrosc. 2019. 10.1007/s00167-019-05634-9. 10.1007/s00167-019-05634-9 31332494

[4] Iversen JV, Krogsgaard MR (2014). Tibial avulsion fracture of the posterior root of the medial meniscus in children. Knee Surg Sports Traumatol Arthrosc.

[5] Hein CN, Deperio JG, Ehrensberger MT, Marzo JM (2011). Effects of medial meniscal posterior horn avulsion and repair on meniscal displacement. Knee.

[6] Feucht MJ, Minzlaff P, Saier T, Lenich A, Imhoff AB, Hinterwimmer S (2015). Avulsion of the anterior medial meniscus root: case report and surgical technique. Knee Surg Sports Traumatol Arthrosc.

[7] Toy JO, Feeley BT, Gulotta LV, Warren RF (2011). Arthroscopic avulsion repair of a pediatric ACL with an anomalous primary insertion into the lateral meniscus. HSS J.

[8] Navarro-Holgado P, Cuevas-Perez A, Aguayo-Galeote MA, Carpintero-Benitez P (2007). Anterior medial meniscus detachment and anterior cruciate ligament tear. Knee Surg Sports Traumatol Arthrosc.

[9] Chen L, Linde-Rosen M, Hwang SC, Zhou J, Xie Q, Smolinski P, Fu FH (2015). The effect of medial meniscal horn injury on knee stability. Knee Surg Sports Traumatol Arthrosc.

[10] Walker PS, Arno S, Bell C, Salvadore G, Borukhov I, Oh C (2015). Function of the medial meniscus in force transmission and stability. J Biomech.

[11] LaPrade CM, Civitarese DM, Rasmussen MT, LaPrade RF (2015). Emerging updates on the posterior cruciate ligament: a review of the current literature. Am J Sports Med.

[12] Zheng J, Zhai W, Li Q, Jia Q, Lin D (2017). A special tear pattern of anterior horn of the lateral meniscus: macerated tear. PLoS One.

[13] Ellman MB, James EW, LaPrade CM, LaPrade RF (2015). Anterior meniscus root avulsion following intramedullary nailing for a tibial shaft fracture. Knee Surg Sports Traumatol Arthrosc.

[14] Marzo JM (2012). Meniscus root avulsion. Clin Sports Med.

[15] Berlet GC, Fowler PJ (1998). The anterior horn of the medical meniscus. An anatomic study of its insertion. Am J Sports Med.

[16] Kale A, Kopuz C, Dikici F, Demir MT, Corumlu U, Ince Y (2010). Anatomic and arthroscopic study of the medial meniscal horns’ insertions. Knee Surg Sports Traumatol Arthrosc.

[17] Allaire R, Muriuki M, Gilbertson L, Harner CD (2008). Biomechanical consequences of a tear of the posterior root of the medial meniscus. Similar to total meniscectomy. J Bone Joint Surg Am.

[18] Schillhammer CK, Werner FW, Scuderi MG, Cannizzaro JP (2012). Repair of lateral meniscus posterior horn detachment lesions: a biomechanical evaluation. Am J Sports Med.

